# Transvenous extraction and reimplantation procedures for quadripolar left ventricular leads with an active fixation side helix

**DOI:** 10.1002/joa3.13134

**Published:** 2024-08-21

**Authors:** Takehiro Nomura, Tsuyoshi Isawa, Shigeru Toyoda, Kennosuke Yamashita, Taku Honda

**Affiliations:** ^1^ Heart Rhythm Center, Department of Cardiovascular Medicine Sendai Kosei Hospital Sendai Japan; ^2^ Department of Cardiovascular Medicine, School of Medicine Dokkyo Medical University Tochigi Japan; ^3^ Department of Cardiovascular Medicine Sendai Kousei Hospital Sendai Japan

**Keywords:** active fixation, cardiac resynchronization therapy, lead extraction, left ventricular lead, reimplantation

## Abstract

Five ASQ extraction cases from our hospital were showed in this list. All leads were completely removed and there were no serious complications. Laser sheaths were used in four of the five cases. In cases 2 and 4, LV leads were successfully reimplanted after the removal of the ASQ, and the original target branches where the ASQ had been implanted remained patent.

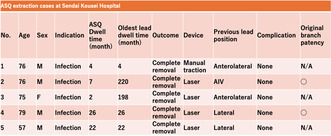

In cardiac resynchronization therapy, the left ventricular (LV) lead position is affected by patient‐specific issues including cardiac vein anomalies, high pacing thresholds, and phrenic nerve stimulation. The Attain Stability Quad (ASQ) (Medtronic, Minneapolis, MN) is a quadripolar LV lead with an active fixation side helix, which allows fixating the lead at the optimal pacing site. However, there are concerns about the feasibility of extracting active fixation leads. A multicenter retrospective study analyzed 26 active fixation LV lead extraction cases and reported that the removal was completed in 24 cases (92.3%),^1^ but the extraction procedure and coronary veins after the active fixation lead extraction have not been sufficiently studied. Therefore, we here report on five cases from Sendai Kousei Hospital.

All procedures were conducted under general anesthesia and transesophageal echocardiographic guidance. The transvenous lead extraction (TLE) procedures were performed as follows: (1) A standard stylet was inserted into the lead and rotated counterclockwise about 30 turns. (2) The proximal portion of the lead was cut off and a locking stylet was inserted. (3) The ASQ lead was attempted to be removed with simple manual traction. (4) If the manual traction was unsuccessful, we attempted to extract the lead using a Glidelight™ (Philips, Amsterdam, the Netherlands) excimer laser sheath.

Table 1 lists five ASQ extraction cases from Sendai Kousei Hospital. All TLEs were performed due to a device infection, and the dwell times were 2, 4, 7, 22, and 26 months, respectively. Three patients were referred from other hospitals. Infections occurred in two of the five patients after upgrading the pacemakers to cardiac resynchronization therapy with defibrillators (CRTDs), with the oldest lead having a dwell time of 16 and 18 years, respectively. All leads were completely removed and there were no serious complications. A Glidelight™ was used in four of the five cases. Tissue ablation around the venous entry site of the lead followed by manual traction allowed the extraction of the ASQ lead in cases 2, 4, and 5. Ablation in the superior vena cava was needed only in a patient with hemodialysis (case 3). Furthermore, in cases 2 and 4, LV leads were reimplanted after the removal of the ASQ, and the original target branches remained patent. We here present those cases, cases 2 and 4, in detail.

**TABLE 1:** ASQ extraction cases at Sendai Kousei Hospital.

No.	Age	Sex	Indication	ASQ dwell time (month)	Oldest lead dwell time (month)	Outcome	Device	Previous lead position	Complication	Original branch patency
1	76	M	Infection	4	4	Complete removal	Manual traction	Anterolateral	None	N/A
2	76	M	Infection	7	220	Complete removal	LS	AIV	None	Patent
3	75	F	Infection	2	198	Complete removal	LS	Anterolateral	None	N/A
4	79	M	Infection	26	26	Complete removal	LS	Lateral	None	Patent
5	57	M	Infection	22	22	Complete removal	LS	Lateral	None	N/A

Abbreviations: AIV, anterior interventricular vein; ASQ, Attain Stability Quad™; CV, coronary vein.

Case 2, a 76‐year‐old man, had undergone a single‐chamber pacemaker implantation for slow atrial fibrillation 18 years prior. He had experienced coronary artery bypass grafting, mitral valve annuloplasty, and a CRTD implantation 7 months before the TLE. The previous LV lead was implanted in the anterior interventricular vein (AIV) (Figure 1A,B) due to a tortuous lateral vein. He developed a pocket infection and was referred to our hospital. A TLE was performed, and the LV lead was easily removed after ablating the tissue around the venous entry site (Figure 1C). The right ventricular lead with a dwell time of 18 years was also successfully extracted. The AIV after the TLE was patent (Figure 1D). A CRTD was successfully reimplanted and we managed to implant the LV lead in a tortuous lateral vein using a “balloon occlusive delivery” (Figure 1E–H). An ASQ was selected for the reimplantation due to the likelihood of occluded target branches and limited lead positioning options, as its active fixation could lead to better pacing thresholds and pacing sites.

**FIGURE 1:**
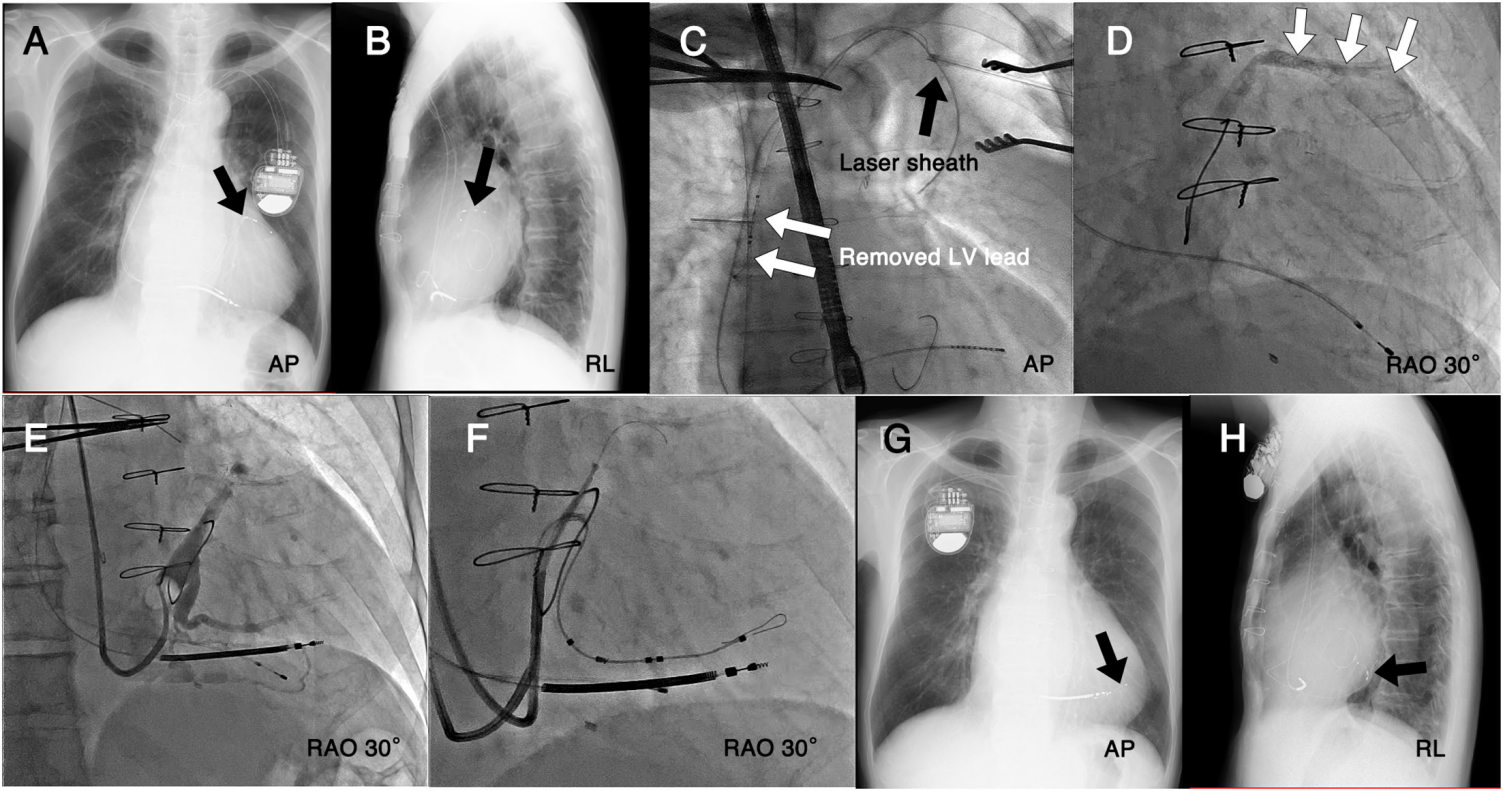
Images showing the extraction procedure of an Attain Stability Quad™ (ASQ) lead with a dwell time of 7 months and the reimplantation of a left ventricular (LV) lead. The original left ventricular (LV) lead was implanted in the anterior interventricular vein (AIV) (arrow) (A, B). The lead was easily removed after ablating the tissue around the venous entry site (C), and the AIV was patent (D). A new ASQ lead was successfully reimplanted in a tortuous lateral vein using the “balloon occlusive delivery” technique (E–H).

Case 4, a 76‐year‐old man with ischemic cardiomyopathy, had a CRTD implantation (Figure 2A) 26 months before the TLE, but he developed a device infection. When the ASQ lead was rotated counterclockwise, the side helix rotation was visually confirmed (Figure 2B,C), but it could not be removed by manual traction alone. It was easily removed after a 12‐French Glidelight™ was used to ablate the tissue around the venous entry site (Figure 2D). After 3 weeks of antimicrobial therapy, a CRTD was reimplanted. The lateral vein remained patent and the lead could be reimplanted (Figure 2E,F). However, a posterolateral branch was finally selected because the pacing thresholds increased to ≥3.0 V/0.4 ms, which were higher than those before the TLE, and the LV electric delay was also longer in the posterolateral area (Figure 2G,H).

**FIGURE 2:**
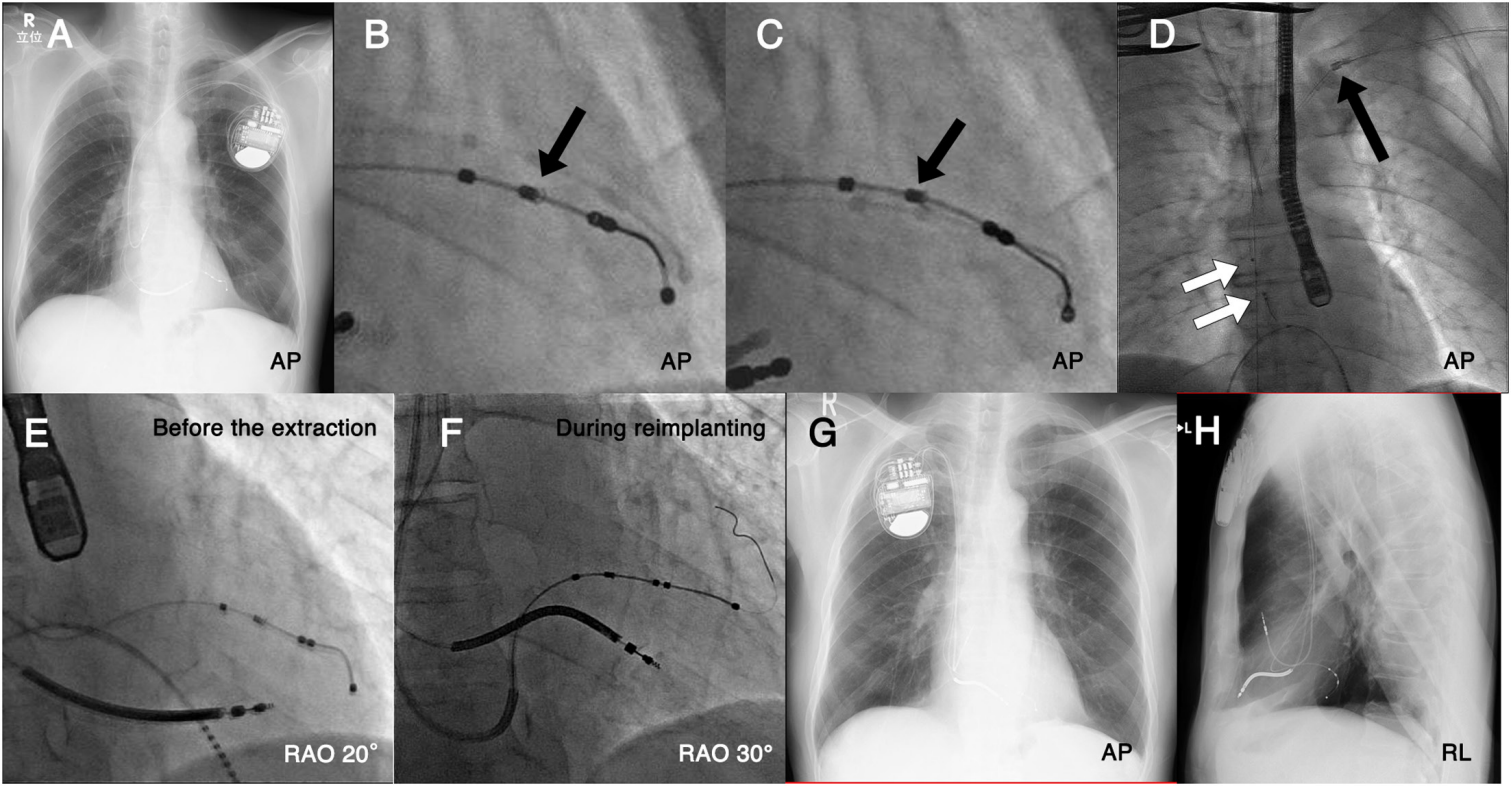
Images showing the extraction procedure of an Attain Stability Quad™ (ASQ) lead with a dwell time of 26 months and the reimplantation of a left ventricular (LV) lead. The previous ASQ lead was implanted in the lateral vein (A). First, the ASQ lead was rotated counterclockwise, and the rotation of the side helix was visually confirmed (B, C). It could be easily removed once the 12‐French Glidelight™ was advanced into the left subclavian vein (D). The lateral vein where the previous ASQ lead had been placed remained patent and the new ASQ lead could be reimplanted (E, F). However, a posterolateral branch was finally selected because the pacing threshold was higher than that before the extraction, and the intrinsic LV electric delay was longer in the lateral vein (G, H).

In our experience, ASQ leads with short dwell times (median 7, range 2–26 months) could be safely removed. Four of five leads required tissue ablation, but all could be removed by manual traction once the adhesions were detached at sites other than the coronary vein. Similar to our report, it has been reported that side helix bipolar leads were successfully removed using only simple traction 63 months post‐implantation.^2^ Conversely, one case report noted that an ASQ lead implanted for 18 months was difficult to remove due to adhesions at the helix site.^3^ In addition, an attain stability in a tortuous branch demonstrated resistance to transvenous extraction, even after 1 year in another single case report.^4^ It might be better to avoid implanting the ASQ in very tortuous branches.

In our two cases where the LV lead was reimplanted, the vessels remained patent. To our knowledge, there are no reports on the post‐removal course of attain stability leads. However, a single‐center study described an LV lead reimplantation after extracting an active fixation lead StarFix™.^5^ StarFix provides additional support within the CS via deployable lobes but is no longer available on the market. The study found that three of four StarFix™ extractions resulted in occluded target branches, with the remaining case having an occluded main coronary sinus, preventing reimplantation in the same branch. This suggests possible differences between StarFix and ASQ. However, in our experience, the pacing threshold in the original target branch was elevated. It was unclear whether a reimplantation in the same branch would be feasible in clinical practice. In the present study, the ASQs were extracted without any complications. However, further research is necessary on extractions of ASQ leads with longer dwell times. Additionally, further investigation is needed not only on the active fixation lead extractions but also on LV lead reimplantations after removal.

## FUNDING INFORMATION

This research did not receive any specific grant from funding agencies in the public, commercial, or not‐for‐profit sectors.

## CONFLICT OF INTEREST STATEMENT

All the authors have no conflicts to disclose.

## ETHICS STATEMENT

The ethical committee of Sendai Kousei Hospital waived the requirement for obtaining ethical approval because this research was neither a clinical study nor an animal experiment.

## PATIENT CONSENT STATEMENT

Written informed consent was obtained from the patient for publication of this report and accompanying images.

## CLINICAL TRIAL REGISTRATION

Not Applicable.

## PERMISSION TO REPRODUCE MATERIAL FROM OTHER SOURCES

This manuscript does not contain any material requiring permission for use.

## Data Availability

Raw data were generated at Sendai Kousei Hospital. Derived data supporting the findings of this study are available from the corresponding author Takehiro Nomura upon request.
